# Supplementary figures and images for: Green Biogenic of Silver Nanoparticles Using Polyphenolic Extract of Olive Leaf Wastes with Focus on Their Anticancer and Antimicrobial Activities
Source: Plants (Basel). 2023 Mar 22;12(6):1410. doi: 10.3390/plants12061410 (PMC10057938; doi:10.3390/plants12061410)

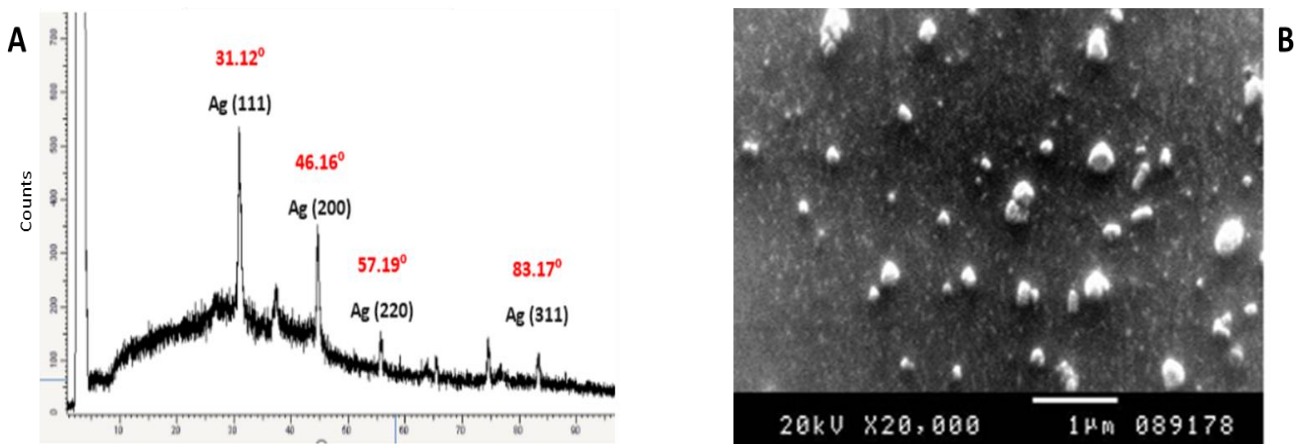

Figure S1. (A) XRD spectrum, (B) SEM micrographs of OLAgnPs biosynthesized by OLWE

Supplement: Supplementary file 1 [file plants-12-01410-s001.zip › plants-2250001-supplementary.pdf]
